# The Efficiency of Commercial Immunodiagnostic Assays for the Field Detection of *Schistosoma japonicum* Human Infections: A Meta-Analysis

**DOI:** 10.3390/pathogens11070791

**Published:** 2022-07-13

**Authors:** Zhongqiu Mei, Shan Lv, Liguang Tian, Wei Wang, Tiewu Jia

**Affiliations:** 1 National Institute of Parasitic Diseases, Chinese Center for Disease Control and Prevention, Key Laboratory of National Health Commission on Parasite and Vector Biology, WHO Collaborating Center for Tropical Diseases, Shanghai 200025, China; meizq1001@163.com (Z.M.); lvshan@nipd.chinacdc.cn (S.L.); tianlg@nipd.chinacdc.cn (L.T.); 2 Key Laboratory of National Health Commission on Parasitic Disease Prevention and Control, Jiangsu Provincial Key Laboratory on Parasites and Vector Control Technology, Jiangsu Institute of Parasitic Diseases, Wuxi 214064, China

**Keywords:** *Schistosoma japonicum*, immunodiagnosis, diagnostic efficiency, indirect hemagglutination test, enzyme-linked immunosorbent assay, dipstick dye immunoassay, meta-analysis

## Abstract

Although great strides have been achieved, schistosomiasis japonica remains a major public health concern in China. Immunodiagnostics have been widely accepted as the first choice in large-scale screening of *Schistosoma japonicum* human infections, and indirect hemagglutination test (IHA), enzyme-linked immunosorbent assay (ELISA), and dipstick dye immunoassay (DDIA) are currently the three most common immunological tests for the diagnosis of *S. japonicum* human infections in China. This meta-analysis aimed to comprehensively assess the performance of IHA, ELISA, and DDIA for the field diagnosis of *S. japonicum* human infections. A total of 37 eligible publications were enrolled in the final analysis, including 29 Chinese publications and 8 English publications. No significant heterogeneities were detected among the studies reporting ELISA (*I*^2^ = 88%, *p* < 0.05), IHA (*I*^2^ = 95%, *p* < 0.05), or DDIA (*I*^2^ = 84%, *p* < 0.05). DDIA showed the highest pooled sensitivity (90.8%, 95% *CI*: 84.6% to 94.7%) and IHA presented the highest pooled specificity for detection of *S. japonicum* human infections (71.6%, 95% *CI*: 65.9% to 76.7%). Summary receiver operating characteristic (SROC) curve analysis showed that IHA exhibited the highest area under the SROC curve (AUC) (0.88, 95% *CI*: 0.85 to 0.9), and ELISA presented the lowest AUC (0.85, 95% *CI*: 0.82 to 0.88). Deeks’ funnel plots indicated no publication bias. IHA presented the highest sensitivity in medium-endemicity regions and the highest specificity for diagnosis of *S. japonicum* human infections in low-endemicity regions, and ELISA showed the highest diagnostic sensitivity in high-endemicity regions and the highest specificity in medium-endemicity regions, while DDIA exhibited the highest diagnostic sensitivity in high-endemicity regions and the highest specificity in low-endemicity regions. IHA and DDIA presented a higher efficiency for the diagnosis of *S. japonicum* human infections in marshland and lake regions than in hilly and mountainous regions, while ELISA showed a comparable diagnostic sensitivity between in marshland and lake regions and hilly and mountainous regions (88.3% vs. 88.6%), and a higher specificity in marshland and lake regions than in hilly and mountainous regions (60% vs. 48%). Our meta-analysis demonstrates a comparable diagnostic accuracy of IHA, ELISA, and DDIA for *S. japonicum* human infections, and the diagnostic sensitivity and specificity of IHA, ELISA, and DDIA vary in types and infection prevalence of endemic regions. DDIA combined with IHA is recommended as a tool for screening chemotherapy targets and seroepidemiological surveys during the stage moving towards schistosomiasis elimination in China. Further studies to examine the effectiveness of combinations of two or three immunological tests for diagnosis of *S. japonicum* human infections are warranted.

## 1. Introduction

Schistosomiasis is a neglected global tropical parasitic disease which affects more than 140 million individuals and causes approximately 200,000 annual deaths worldwide [1]. The recent great strides urged the ambitious goal set for elimination of schistosomiasis as a public health problem in all disease-endemic countries in the world by 2030; however, great challenges have been identified to achieve this ambitious goal [2,3]. Unfortunately, the global pandemic of COVID-19 poses negative effects on global schistosomiasis elimination programs, adding challenges to achieve the goal of a schistosomiasis-free world [4,5,6].

Schistosomiasis in China, Indonesia, and the Philippines is caused by infections with *Schistosoma japonicum* as the predominant species [7]. China was once highly endemic for *S. japonicum* and suffered from the highest burden of schistosomiasis in the world [8]. After the national schistosomiasis control program was initiated in the 1950s, great successes have been achieved, and schistosomiasis had been eliminated as a public health problem in China according to the World Health Organization-defined criteria as of 2015 [9]. Nevertheless, multiple challenges are identified to completely wipe out the “God of Plague” in the country [10].

Diagnosis is central to the schistosomiasis control program, which is necessary for identification of chemotherapy targets, assessment of chemotherapy efficacy, as well as planning, implementation, and evaluation of the effectiveness of the schistosomiasis control program [11]. Currently, detection of parasite eggs or juvenile parasites with parasitological techniques remains the gold standard for the definitive diagnosis of schistosomiasis japonica; however, these tools suffer from problems of time-consuming procedures, low participation rate, and high false negative rate in low-endemicity regions [12]. A large number of emerging molecular assays have shown potential in precise early diagnosis of schistosomiasis japonica; however, these DNA- or RNA-based assays suffer from problems of laboratory-intensive procedures, high costs, and requirements of professional healthcare education, and there have been no commercial molecular kits available for the clinical diagnosis of *S. japonicum* human infections until now [13,14,15]. Immunodiagnostics, which are rapid and easy to perform, are currently the most efficacious and practical means for diagnosis of human schistosomiases based on the detection of infection-specific antibodies and have been widely accepted as the first choice in large-scale screening of *S. japonicum* human infections, seroepidemiological surveys, and assessment of the effectiveness of the schistosomiasis control program in China [16], although the performance of immunodiagnostic assays for early detection of *S. japonicum* human infections remains to be improved [17,18].

Currently, indirect hemagglutination test (IHA), enzyme-linked immunosorbent assay (ELISA), and dipstick dye immunoassay (DDIA) are the three most common immunological tests for the diagnosis of *S. japonicum* human infections in China [17,18]. During the stage moving towards elimination of schistosomiasis, the option of immunodiagnostics is a critical part of the national schistosomiasis elimination program in China [19]. However, there have been no pooled estimates of the sensitivity and specificity of commercial immunological tests for the diagnosis of *S. japonicum* human infections in endemic foci with different epidemic types and levels [20,21,22]. Based on data from public databases, this study aimed to comprehensively assess the performance of the three most common commercial immunodiagnostic assays, including IHA, ELISA, and DDIA, for the diagnosis of *S. japonicum* human infections in endemic foci of China, so as to provide insights into the option of immunodiagnostics for the national schistosomiasis elimination program in China.

## 2. Methods

### 2.1. Literature Search

A joint search was performed in international and national electronic databases, including Web of Science, PubMed, Scopus, Google Scholar, and Chinese electronic databases CNKI (https://www.cnki.net/; accessed on 7 April 2022), Wanfang Data (https://www.wanfangdata.com.cn; accessed on 7 April 2022) and VIP (http://www.cqvip.com/; accessed on 7 April 2022) using the mesh terms ((schistosom *) OR (bilharzia *)) AND ((serologic * test) OR (immunological test) OR (ELISA) OR (IHA) OR (DDIA)) AND ((stool examination) OR (Kato-Katz) OR (miracidium hatching)), to retrieve English and Chinese publications pertaining to the diagnosis of *S. japonicum* human infections with IHA, ELISA, or DDIA. The time of search was defined as from 1980 to 2021. In addition, the post-text references of retrieved publications were read, and grey literatures, such as institutional annual reports, proceedings, and collections, were artificially searched to track all possibly related studies.

### 2.2. Inclusion and Exclusion Criteria

We defined the following inclusion criteria: (1) there were parasitological tests as the gold standard for diagnosis of *S. japonicum* human infections in the studies, such as Kato-Katz technique or miracidium hatching test; (2) there were one or multiple uses of thee three immunological tests (IHA, ELISA, DDIA) in the study; (3) the immunological assay had been commercial and standardized used in the schistosomiasis-endemic foci; (4) there were detailed numbers to calculate true positives (TP), false positives (FP), true negatives (TN), and false negatives (FN) in the study; (5) immunological assays were used for detection of *S. japonicum* human infections in the study; (6) immunological tests were performed independently and blindly; and (7) full-text files were available. All studies that met the following criteria were excluded from the analysis: (1) no parasitological tests; (2) immunological assays were used for detection of *S. japonicum* infections in animals; (3) case-control studies; (4) no detailed numbers to calculate TP, FP, TN, or FN; (5) review articles or meeting reports; (6) the study sample size was less than 50; or (7) full-text publications were unavailable.

### 2.3. Data Extraction

Following joint search in electronic databases, repeated publications were excluded, and the title and abstract of screened literatures were carefully read. Then, eligible studies were identified based on defined inclusion and exclusion criteria. Each grey literature was reviewed carefully based on the defined inclusion and exclusion criteria. All data were managed using the software Microsoft Excel 2010 (Microsoft Inc., Redmond, WA, USA). The title, authors, year of publication, serological tests, participants’ age, epidemic types, endemicity, the gold standard used for detection of *S. japonicum* human infections, TP, FP, TN, and FN were extracted by two independent investigators. If there was a disagreement, an additional investigator was introduced, and the final decision was made by the third investigator.

### 2.4. Asymmetry Test

The potential presence of publication bias was evaluated using the Deeks’ funnel plot created with the software Stata version 14.0 (Stata Corporation Lakeway, TX, USA) [23]. An asymmetrical funnel plot indicated the presence of publication bias.

### 2.5. Meta-Analysis

Since the “threshold effect” has been recognized as a notable source of heterogeneity for diagnostic tests [24], the presence of heterogeneity caused by the “threshold effect” was tested using the software Stata version 14.0. Then, the heterogeneity among studies was examined using the software OpenMeta [analyst] version 3.3 prior to pooled estimates. If *I*^2^ statistic was <50% and *p* > 0.05 in the *Q* test, no significant heterogeneity was identified among studies, and a fixed-effect model was employed for pooled estimates; otherwise, a random-effect model was used. A summary receiver operating characteristic (SROC) curve was plotted using the software Stata version 14.0 in order to compare the diagnostic accuracy of three immunological assays, and the area under the SROC curve (AUC) was calculated. A higher AUC indicated a greater diagnostic accuracy.

Next, subgroup analyses were performed according to the test of heterogeneity, to examine the effects of the endemicity and epidemic types of schistosomiasis on the performance of IHA, ELISA, and DDIA for detection of *S. japonicum* human infections.

In this study, the epidemic types were classified into marshland and lake regions, plain regions with waterway networks and hilly and mountainous regions [25], and the epidemic level was classified based on the prevalence of *S. japonicum* human infections: high endemicity, infection prevalence of 10% and higher; medium endemicity, infection prevalence of <10% and no less than 5%; and low endemicity, infection prevalence of <5% [26].

## 3. Results

### 3.1. Study Characteristics

**Figure 1:**
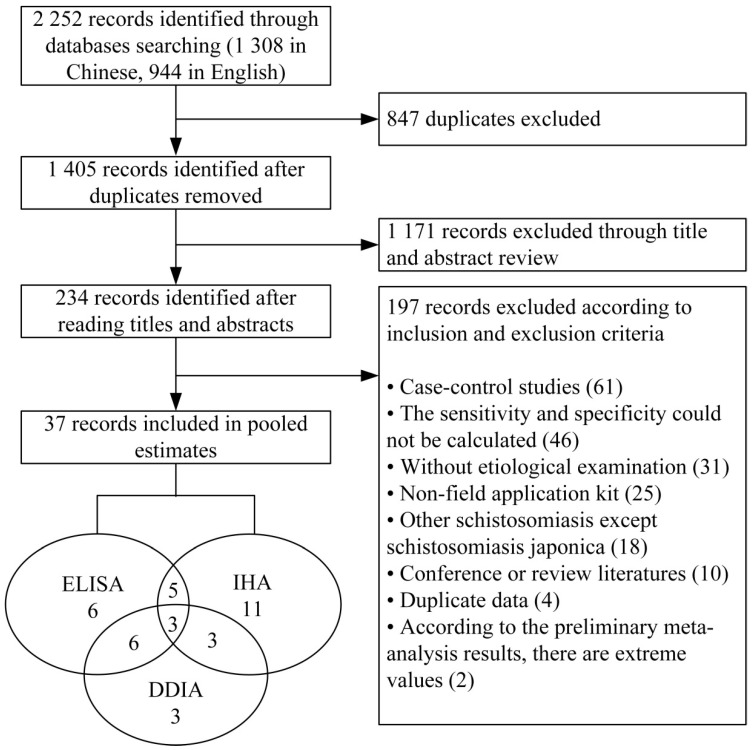
Flow chart of publication selection.

A total of 2252 publications were screened, including 1308 Chinese publications and 944 English publications, and 37 eligible publications that met the inclusion and exclusion criteria were enrolled in the final analysis, including 29 Chinese publications and 8 English publications (Figure 1). Table 1 demonstrates the characteristics of all included studies.

### 3.2. Meta-Analysis

**Figure 2:**
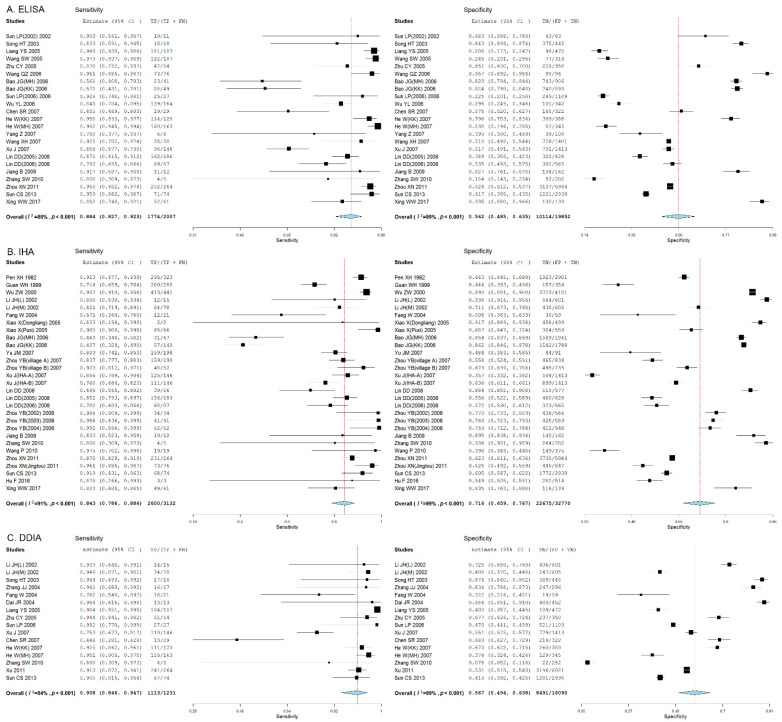
Forest plots show the pooled sensitivity and specificity of ELISA, IHA, and DDIA for the diagnosis of *Schistosoma japonicum* human infections. (**A**) Forest plot of the pooled sensitivity and specificity of ELISA for the diagnosis of *S. japonicum* human infections; (**B**) Forest plot of the pooled sensitivity and specificity of IHA for the diagnosis of *S. japonicum* human infections; (**C**) Forest plot of the pooled sensitivity and specificity of DDIA for the diagnosis of *S. japonicum* human infections.

We found that the threshold effect contributed 12%, 1%, and 3% to the heterogeneity for ELISA, IHA, and DDIA, respectively. The test of heterogeneity revealed significant heterogeneities among the studies reporting ELISA (*I*^2^ = 88%, *p* < 0.05), IHA (*I*^2^ = 95%, *p* < 0.05), and DDIA (*I*^2^ = 84%, *p* < 0.05), and a random-effect model was therefore employed for pooled estimates. DDIA showed the highest pooled sensitivity (90.8%, 95% *CI*: 84.6% to 94.7%) and IHA presented the highest pooled specificity for detection of *S. japonicum* human infections (71.6%, 95% *CI*: 65.9% to 76.7%) (Figure 2).

### 3.3. Comparison of the Diagnostic Accuracy of Three Immunodiagnostic Assays

**Figure 3:**
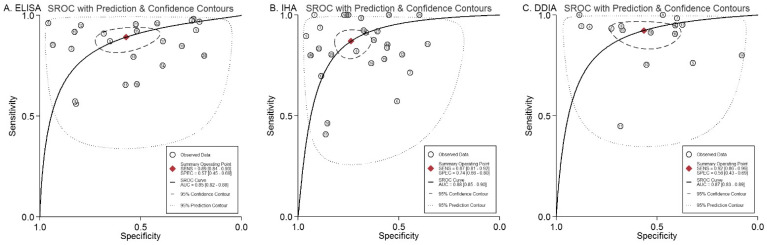
SROC curves for the diagnostic accuracy of ELISA, IHA, and DDIA for the detection of *Schistosoma japonicum* human infections. (**A**) SROC curve for the diagnostic accuracy of ELISA for the detection of *S. japonicum* human infections; (**B**) SROC curve for the diagnostic accuracy of IHA for the detection of *S. japonicum* human infections; (**C**) SROC curve for the diagnostic accuracy of DDIA for the detection of *S. japonicum* human infections.

SROC curve analysis showed that IHA exhibited the highest AUC (0.88, 95% *CI*: 0.85 to 0.9), and ELISA presented the lowest AUC (0.85, 95% *CI*: 0.82 to 0.88) (Figure 3), indicating that IHA has the highest accuracy for the diagnosis of *S. japonicum* human infections.

### 3.4. Publication Bias

**Figure 4:**
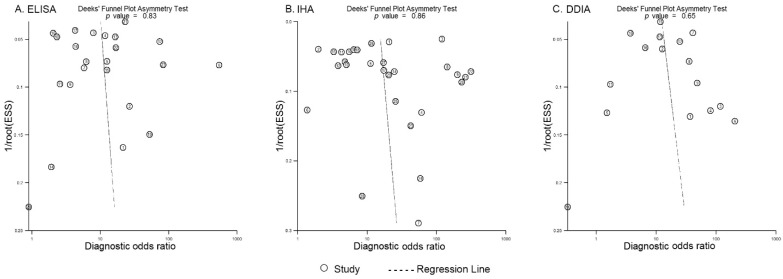
Deeks’ funnel plots of studies reporting ELISA, IHA, and DDIA. (**A**) Deeks’ funnel plot of studies reporting ELISA; (**B**) Deeks’ funnel plot of studies reporting IHA; (**C**) Deeks’ funnel plot of studies reporting DDIA. An asymmetrical funnel plot indicates the presence of publication bias.

Deeks’ funnel plots were created to evaluate the publication bias of included studies. All three funnel plots were found to be almost symmetrical (Figure 4), and asymmetry test revealed no statistical significances (*p* > 0.05), indicating no publication bias.

### 3.5. Subgroup Analysis

**Figure 5:**
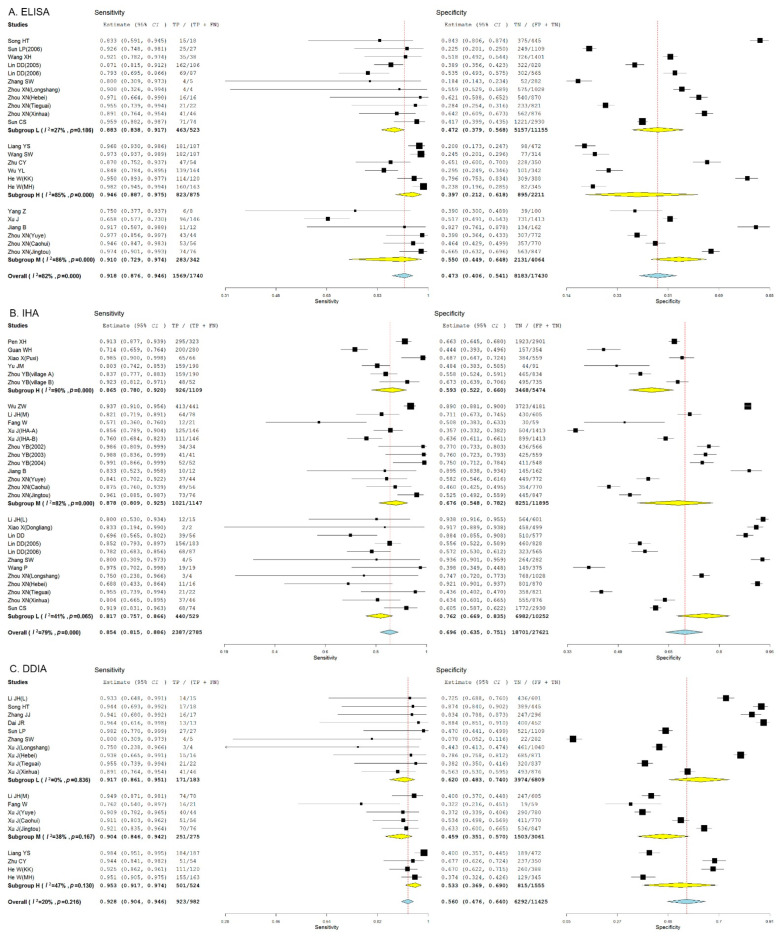
Forest plots show the pooled sensitivity and specificity of ELISA, IHA, and DDIA for the diagnosis of *Schistosoma japonicum* human infections in regions with different endemic levels. (**A**) Forest plot of the pooled sensitivity and specificity of ELISA for the diagnosis of *Schistosoma japonicum* human infections in regions with different endemic levels; (**B**) Forest plot of the pooled sensitivity and specificity of IHA for the diagnosis of *Schistosoma japonicum* human infections in regions with different endemic levels; (**C**) Forest plot of the pooled sensitivity and specificity of DDIA for the diagnosis of *Schistosoma japonicum* human infections in regions with different endemic levels.

ELISA presented the highest sensitivity in high-endemicity regions (94.6%, 95% *CI*: 88.7% to 97.5%), and the highest specificity for diagnosis of *S. japonicum* human infections in medium-endemicity regions (55%, 95% *CI*: 44.9% to 64.8%) (Figure 5A), and IHA showed the highest diagnostic sensitivity in medium-endemicity regions (87.8%, 95% *CI*: 80.9% to 92.5%) and the highest diagnostic specificity in low-endemicity regions (76.2%, 95% *CI*: 66.9% to 83.5%) (Figure 5B), while DDIA exhibited the highest diagnostic sensitivity in high-endemicity regions (95.3%, 95% *CI*: 91.7% to 97.4%), and the highest diagnostic specificity in low-endemicity regions (62%, 95% *CI*: 48.3% to 74%) (Figure 5C). Overall, DDIA presented the highest pooled sensitivity (92.8%, 95% *CI*: 90.4% to 94.6%), and IHA showed the highest pooled specificity for diagnosis of *S. japonicum* human infections (69.6%, 95% *CI*: 63.5% to 75.1%).

**Figure 6:**
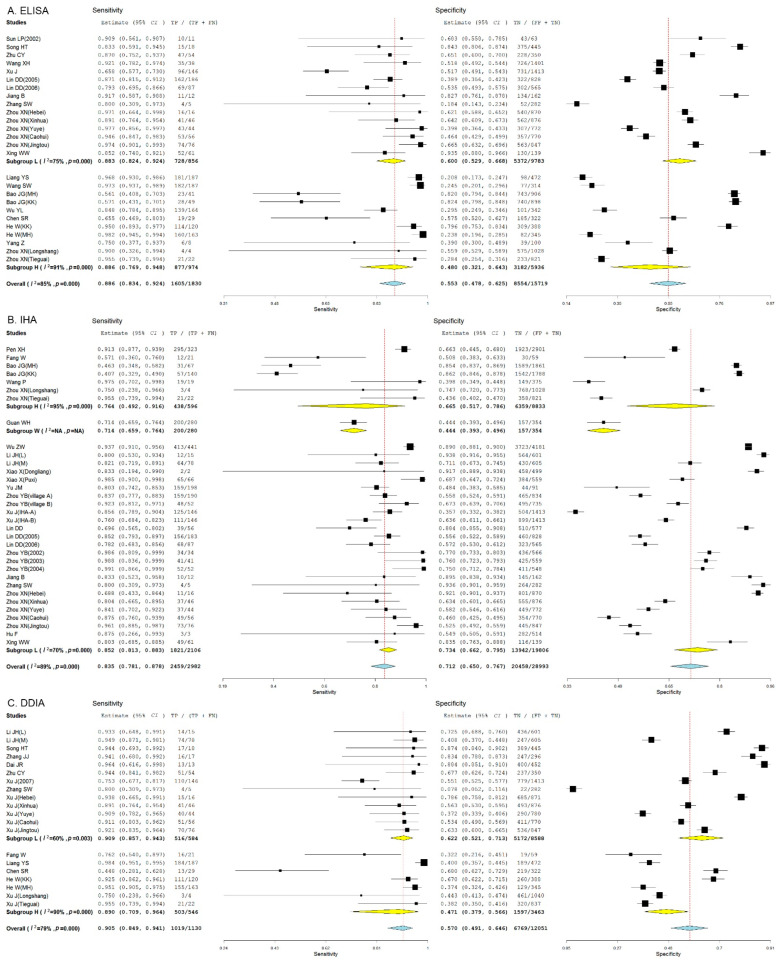
Forest plots show the pooled sensitivity and specificity of ELISA, IHA, and DDIA for the diagnosis of *Schistosoma japonicum* human infections in endemic foci with different epidemic types. (**A**) Forest plot of the pooled sensitivity and specificity of ELISA for the diagnosis of *Schistosoma japonicum* human infections in endemic foci with different epidemic types; (**B**) Forest plot of the pooled sensitivity and specificity of IHA for the diagnosis of *Schistosoma japonicum* human infections in endemic foci with different epidemic types; (**C**) Forest plot of the pooled sensitivity and specificity of DDIA for the diagnosis of *Schistosoma japonicum* human infections in endemic foci with different epidemic types.

ELISA presented a comparable sensitivity between in hilly and mountainous regions (88.6%, 95% *CI*: 76.9% to 94.8%) and marshland and lake regions (88.3%, 95% *CI*: 82.4% to 92.4%), and a higher specificity for diagnosis of *S. japonicum* human infections in marshland and lake regions (60%, 95% *CI*: 52.9% to 66.8%) than in hilly and mountainous regions (48%, 95% *CI*: 32.1% to 64.3%) (Figure 6A), and IHA showed a higher pooled diagnostic sensitivity (85.2%, 95% *CI*: 81.3% to 88.3%) and specificity (73.4%, 95% *CI*: 66.2% to 79.5%) in marshland and lake regions than in hilly and mountainous regions (76.4%, 95% *CI*: 49.2% to 91.6%; 66.5%, 95% *CI*: 51.7% to 78.6%) and plain regions with waterway networks (71.4%, 95% *CI*: 65.9% to 76.4%; 44.4%, 95% *CI*: 39.3% to 49.6%) (Figure 6B), while DDIA exhibited a higher pooled diagnostic sensitivity (90.9%, 95% *CI*: 85.7% to 94.3%) and specificity (62.2%, 95% *CI*: 52.1% to 71.3%) in marshland and lake regions than in hilly and mountainous regions (89%, 95% *CI*: 70.9% to 96.4%; 47.1%, 95% *CI*: 39.7% to 56.6%) (Figure 6C). Similarly, DDIA presented the highest pooled sensitivity (90.5%, 95% *CI*: 84.9% to 94.1%) and IHA showed the highest pooled specificity for diagnosis of *S. japonicum* human infections (71.2%, 95% *CI*: 65% to 76.7%).

## 4. Discussion

Precise diagnosis, which is a prerequisite to chemotherapy, is extremely helpful in implementing strategies for the control and elimination of schistosomiasis, which plays a pivotal role in schistosomiasis control programs [64]. Following the concerted efforts for more than seven decades, the epidemiological features of schistosomiasis are characterized by low prevalence and low-infection intensity in China [65,66,67]. Conventional parasitological tools, which remain the gold standard for the diagnosis of schistosomiasis, fail to meet the needs of precise identification of *S. japonicum* human infections in endemic foci of China, because of its high rate of missing diagnosis in low-endemicity regions [15]. To achieve early, precise identification of *S. japonicum* infections, multiple PCR assays have been developed and shown potential for the field detection of *S. japonicum* human infections; however, these assays require specific equipment and are high in costs, making them unlikely to be used for large-scale screening and epidemiological surveys in schistosomiasis-endemic foci [68,69,70]. In addition, loop-mediated isothermal amplification (LAMP) assays were developed for accurate, visualized, and early detection of *S. japonicum* human infections; however, these assays are extremely likely to be contaminated, resulting in false positives [71,72,73]. Recently, amplification recombinase-aided isothermal amplification (RAA) and recombinase polymerase amplification (RPA) assays have been developed for early detection of *S. japonicum* human infections; however, the performance of RAA and RPA assays remains to be investigated for detection of *S. japonicum* infections in large-scale clinical studies [74,75,76,77,78].

Antibody-based immunodiagnostics have been accepted as the first choice for large-scale screening and seroepidemiological surveys of *S. japonicum* human infections [64]. Currently, there are four commercial serological kits used for diagnosis of *S. japonicum* human infections in China, including IHA, ELISA, DDIA, and dot immunogold filtration assay (DIGFA), and IHA, ELISA, and DDIA are the three most common approaches used for schistosomiasis immunodiagnosis because of simple, rapid procedures and low costs [17]. Since the diagnostic effectiveness of IHA, ELISA, and DDIA for schistosomiasis has been reported to vary in endemic foci, a precise and comprehensive assessment of the performance of these serological tests for detection of *S. japonicum* human infections is therefore of great significance to optimize the option of immunodiagnostic assays in various endemic foci of China.

In this study, a total of 37 eligible studies that met the inclusion and exclusion criteria were enrolled in meta-analysis, and no publication bias was detected among studies by the asymmetry test. The highest pooled sensitivity for detection of *S. japonicum* human infections was seen for DDIA, with the lowest for IHA, and the highest pooled specificity was seen IHA, with the lowest for ELISA, which was in agreement with a previous meta-analysis reporting that IHA, ELISA, and DDIA presented the pooled sensitivities of 0.83, 0.87, and 0.90 and specificities of 0.69, 0.60, and 0.62 for diagnosis of schistosomiasis japonica [20]. In addition, SROC curve analysis showed that IHA exhibited the highest accuracy and ELISA presented the lowest accuracy for the diagnosis of *S. japonicum* human infections, which was inconsistent with previous results showing 0.89, 0.96, and 0.92 AUCs for IHA, ELISA, and DDIA [20]. This may be attributed to the difference of included studies. However, our findings are in agreement with two previous meta-analyses reporting a higher accuracy of IHA than ELISA for diagnosis of schistosomiasis japonica [21,22].

Since there are three types of endemic foci in China [25], we performed a subgroup analysis to estimate the pooled sensitivity and specificity for detection of *S. japonicum* human infections in endemic foci with different epidemic types. Our findings showed that IHA and DDIA presented a higher efficiency for the diagnosis of *S. japonicum* human infections in marshland and lake regions than in hilly and mountainous regions; however, ELISA showed a comparable diagnostic sensitivity between in marshland and lake regions and hilly and mountainous regions (88.3% vs. 88.6%), and a higher specificity in marshland and lake regions than in hilly and mountainous regions (60% vs. 48%). All these three immunological tests are antibody-based assays, and the differences in diagnostic sensitivity and specificity are considered to be explained by that the antigens used for preparation of these three immunodiagnostics are derived from *S. japonicum* isolates from the marshland and lake regions.

To compare the performance of three immunological tests for detection of *S. japonicum* human infections in regions with different endemic levels, a subgroup analysis was performed. Our findings showed the highest diagnostic sensitivity of IHA in medium-endemicity regions and the highest specificity in low-endemicity regions, the highest diagnostic sensitivity of ELISA in high-endemicity regions and highest specificity in medium-endemicity regions, and the highest diagnostic sensitivity of DDIA in high-endemicity regions and the highest specificity in low-endemicity regions. Results from a previous meta-analysis showed that the sensitivities of IHA, ELISA, and DDIA were 0.84, 0.76, and 0.94; 0.88, 0.80, and 0.93; and 0.93, 0.81, and 0.93 in high-, medium-, and low-endemicity regions, and the specificities were 0.73, 0.64, and 0.73; 0.59, 0.59, and 0.62; and 0.66, 0.69, and 0.59 in high-, medium-, and low-endemicity regions, respectively [20], which was inconsistent with our data. This may be attributed to the variation of included studies.

To accelerate the achievements of the target for the elimination of schistosomiasis as a public health problem and the interruption of transmission in humans in selected countries by 2030 set out in the WHO road map “Ending the neglect to attain the Sustainable Development Goals: A road map for neglected tropical diseases 2021–2030” [79], a new guideline for the control and elimination of human schistosomiasis was released by WHO in February 2022 [80]. In this new guideline, six evidence-based recommendations were proposed for the control and elimination of human schistosomiasis in disease-affected countries, including diagnostic strategies for assessment of schistosomiasis infection in humans [80]. In this study, we found a diverse diagnostic sensitivity and specificity of IHA, ELISA, and DDIA for detection of *S. japonicum* human infections in different types and infection prevalence of endemic regions of China, and DDIA presented the highest pooled sensitivity, while IHA showed the highest pooled specificity for diagnosis of *S. japonicum* human infections. Large-scale diagnostic tests to compare the performance of IHA, ELISA, and DDIA for detection of *S. japonicum* human infections in same settings are encouraged. Based on successful experiences from the national schistosomiasis control program, China had been supporting schistosomiasis elimination programs in disease-endemic countries along the Belt and Road Initiative [81]. China-made praziquantel and chemical molluscicides have shown effective for schistosomiasis control in African countries [82,83]. Although IHA, ELISA, and DDIA are produced based on the antigens from *S. japonicum* isolates, previous studies have shown the effectiveness of DDIA and IHA for the detection of *S. mekongi*, *S. mansoni*, and *S*. *haematobium* human infections [84,85,86,87]. Further large-scale diagnostic tests to investigate the performance of China-made commercial immunodiagnostic assays for the diagnosis of African schistosomiasis seem justified, with may provide tools to support the elimination of schistosomiasis in African continents. In addition, improvements of China-made immunodiagnostics with antigens from *S. mansoni* and *S*. *haematobium* isolates may improve the sensitivity and specificity for diagnosis of *S. mansoni* and *S*. *haematobium* human infections.

This study has some limitations. First, the immunodiagnostics were provided by different manufacturers; however, we did not compare the diagnostic performance of immunodiagnostics by different manufacturers for schistosomiasis immunodiagnosis, since the suppliers of some immunodiagnostics were not available in publications. Second, we did not perform a subgroup analysis to compare the effectiveness of immunodiagnostic assays among participants with different ages.

In summary, the results of our meta-analysis demonstrate a comparable diagnostic accuracy of IHA, ELISA, and DDIA for *S. japonicum* human infections, and the diagnostic sensitivity and specificity of IHA, ELISA, and DDIA vary in endemic regions with different epidemic types and endemic levels. In addition, DDIA presents the highest pooled sensitivity and IHA shows the highest pooled specificity for diagnosis of *S. japonicum* human infections. Since schistosomiasis is currently low in prevalence and infection intensity in China, DDIA in combination with IHA is recommended as a tool for screening chemotherapy targets and seroepidemiological surveys. Further studies to examine the effectiveness of combinations of two or three immunological tests for diagnosis of *S. japonicum* human infections are warranted.

## Figures and Tables

**Table 1:** Subject characteristics of the included studies.

Publication Year	Subjects’ Age (Years)	Degree of Endemicity	Epidemic Types	Immunological Assay	Parasitological Technique	True Positives	False Negatives	True Negatives	False Positives	Reference
1982	>15	High	Hilly and mountainous regions	IHA	Miracidium hatching test (three slides from three stool samples)	295	28	1923	978	[27]
1999	3 to 70	High	Plain regions with waterway networks	IHA	Kato-Katz (two slides from one stool sample)	200	80	157	197	[28]
2000	6 to 60	Medium	Marshland and lake regions	IHA	Kato-Katz	413	28	3723	458	[29]
2002	5 to 56	Low	Marshland and lake regions	IHA	Kato-Katz (three slides from one stool sample)	12	3	564	37	[30]
				DDIA		14	1	436	165	
2002	N/A	Medium	Marshland and lake regions	IHA	Kato-Katz (three slides from one stool sample)	64	14	430	175	[31]
				DDIA		74	4	247	358	
2002	5 to 60	N/A	Marshland and lake regions	ELISA	Miracidium hatching test	10	1	43	20	[32]
2003	6 to 64	Low	Marshland and lake regions	DDIA	Kato-Katz (three slides from one stool sample) and miracidium hatching test	17	1	389	56	[33]
				ELISA		15	3	375	70	
2004	N/A	Low	Marshland and lake regions	DDIA	Kato-Katz	16	1	247	49	[34]
2004	6 to 60	Medium	Hilly and mountainous regions	IHA	Miracidium hatching test	12	9	30	29	[35]
				DDIA		16	5	19	40	
2004	60 to 65	Low	Marshland and lake regions	DDIA	Miracidium hatching test (three slides from three stool samples)	13	0	400	52	[36]
2005	15 to 70	High	Marshland and lake regions	DDIA	Miracidium hatching test (three slides from one stool sample)	51	3	237	113	[37]
				ELISA		47	7	228	122	
2005	10 to 70	High	Hilly and mountainous regions	DDIA	Kato-Katz (three slides from one stool sample)	184	3	189	283	[38]
				ELISA		181	6	98	374	
2005	6 to 65	High	Hilly and mountainous regions	ELISA	Kato-Katz (three slides from one stool sample) and miracidium hatching test	182	5	77	237	[39]
2005	N/A	Low	Hilly and mountainous regions	IHA	Kato-Katz (three slides from one stool sample)	2	0	458	41	[40]
		High				65	1	384	175	
2006	5 to 65	N/A	Hilly and mountainous regions, and marshland and lake regions	ELISA	Kato-Katz (six slides from two stool samples)	73	3	90	4	[41]
				IHA		68	8	94	4	
2006	N/A	High	Hilly and mountainous regions	ELISA	Miracidium hatching test	139	25	101	241	[42]
2006	>5	High, medium, and low	Hilly and mountainous regions	IHA	Miracidium hatching test (three slides from three stool samples)	31	36	1589	272	[43]
					Kato-Katz (two slides from one stool sample)	57	83	1542	246	
				ELISA	Miracidium hatching test (three slides from three stool samples)	23	18	743	163	
					Kato-Katz (two slides from one stool sample)	28	21	740	158	
2006	6 to 65	Low	Marshland and lake regions, plain regions with waterway networks, and hilly and mountainous regions	ELISA	Miracidium hatching test (one slide from one stool sample)	25	2	249	860	[44]
				DDIA		27	0	521	588	
2007	>5	High, medium, and low	Hilly and mountainous regions	ELISA	Miracidium hatching test (one slide from one stool sample) and Kato-Katz (four slides from one stool sample)	19	10	185	137	[45]
				DDIA		13	16	219	103	
2007	10 to 70	High	Hilly and mountainous regions	DDIA	Kato-Katz (three slides from one stool sample)	111	9	260	128	[46]
				ELISA		114	6	309	79	
				DDIA	Miracidium hatching test (one slide from one stool sample)	155	8	129	216	
				ELISA		160	3	82	263	
2007	6 to 65	Medium	Marshland and lake regions	DDIA	Miracidium hatching test (three slides from one stool sample) and Kato-Katz (three slides from one stool sample)	110	36	779	634	[47]
				ELISA		96	50	731	682	
				IHA-A		125	21	504	909	
				IHA-B		111	35	899	514	
2007	6 to 65	Medium	Hilly and mountainous regions	ELISA	Miracidium hatching test (three slides from three stool samples)	6	2	39	61	[48]
2007	5 to 75	Village A: high	Marshland and lake regions	IHA	Kato-Katz (three slides from one stool sample)	159	31	465	369	[49]
		Village B: medium		IHA		48	4	495	240	
2007	N/A	High	Marshland and lake regions	IHA	Kato-Katz (seven slides from one stool sample) and miracidium hatching test	159	39	44	47	[50]
2007	6 to 65	Low	Hilly and mountainous regions	ELISA	Kato-Katz (four slides from one stool sample)	35	3	726	675	[51]
2008	N/A	N/A	Marshland and lake regions	IHA	Kato-Katz (twelve slides from two stool samples)	39	17	510	67	[52]
2008	6 to 65	Medium	Hilly and mountainous regions	IHA	Kato-Katz (three slides from one stool sample)	34	0	436	130	[53]
				IHA		41	0	425	134	
				IHA		52	0	411	137	
2008	>5	High	Marshland and lake regions	IHA	Kato-Katz (six slides from two stool samples)	156	27	460	368	[54]
				IHA		68	19	323	242	
2008	>5	High	Marshland and lake regions	ELISA	Kato-Katz (six slides from two stool samples)	162	24	322	506	[55]
				ELISA		69	18	302	263	
2009	11 to 46	Medium	Marshland and lake regions	IHA	Kato-Katz (three slides from one stool sample)	10	2	145	17	[56]
				ELISA		11	1	134	28	
2010	6 to 65	Low	Marshland and lake regions	IHA	Miracidium hatching test (three slides from one stool sample)	4	1	264	18	[57]
				DDIA		4	1	22	260	
				ELISA		4	1	52	230	
2010	5 to 80	Low	Hilly and mountainous regions	IHA	Kato-Katz (nine slides from three stool samples) and miracidium hatching test	19	0	149	226	[58]
2011	6 to 65	Medium and low	Marshland and lake regions, and hilly and mountainous regions	DDIA	Kato-Katz (three slides from one stool sample) and miracidium hatching test	241	23	3196	2825	[59]
2011	6 to 65	Medium and low	Marshland and lake regions, and hilly and mountainous regions	IHA	Kato-Katz (three slides from one stool sample) and miracidium hatching test	231	33	3370	2254	[60]
				ELISA		252	12	3137	2847	
2013	6 to 65	Low	Marshland and lake regions, and hilly and mountainous regions	IHA	Kato-Katz (three slides from one stool sample) and miracidium hatching test	68	6	1772	1158	[61]
				DDIA		67	7	1201	1729	
				ELISA		71	3	1221	1709	
2016	>5	N/A	Marshland and lake regions	IHA	Kato-Katz (twenty seven slides from three stool samples)	3	0	282	232	[62]
2017	N/A	N/A	Marshland and lake regions	IHA	Kato-Katz (three slides from one stool sample) and miracidium hatching test	49	12	116	23	[63]
				ELISA		52	9	130	9	

N/A, subjects’ age or the degree of the endemicity was not reported in the study.

## Data Availability

All data presented in this study are available upon request by contact with the corresponding authors.

## References

[1] Verjee M.A. (2019). Schistosomiasis: Still a Cause of significant morbidity and mortality. Res. Rep. Trop. Med..

[2] Giboda M., Bergquist R., Utzinger J. (2022). Schistosomiasis at the crossroad to elimination: Review of eclipsed research with emphasis on the post-transmission agenda. Trop. Med. Infect. Dis..

[3] Deol A.K., Fleming F.M., Calvo-Urbano B., Walker M., Bucumi V., Gnandou I., Tukahebwa E.M., Jemu S., Mwingira U.J., Alkohlani A. (2019). Schistosomiasis—Assessing progress toward the 2020 and 2025 global goals. N. Engl. J. Med..

[4] Li G., Xu D., Hu Y., Xu M., Zhang L., Du X., Zhang L., Sun C., Xie Y., Tan X. (2022). Impact of the coronavirus disease 2019 lockdown on *Schistosoma* host *Oncomelania hupensis* density in Wuhan. Acta Trop..

[5] Toor J., Adams E.R., Aliee M., Amoah B., Anderson R.M., Ayabina D., Bailey R., Basáñez M.G., Blok D.J., Blumberg S. (2021). Predicted impact of COVID-19 on neglected tropical disease programs and the opportunity for innovation. Clin. Infect. Dis..

[6] Mantica G., Martini M., Riccardi N. (2021). The possible impact of SARS-CoV-2 on neglected tropical diseases in Europe: The out of spotlights emerging of schistosomiasis. J. Prev. Med. Hyg..

[7] McManus D.P., Dunne D.W., Sacko M., Utzinger J., Vennervald B.J., Zhou X.N. (2018). Schistosomiasis. Nat. Rev. Dis. Primers.

[8] Hong Z., Li L., Zhang L., Wang Q., Xu J., Li S., Zhou X.N. (2022). Elimination of schistosomiasis japonica in China: From the One Health perspective. China CDC Wkly..

[9] Xu J., Li S.Z., Zhang L.J., Bergquist R., Dang H., Wang Q., Lv S., Wang T.P., Lin D.D., Liu J.B. (2020). Surveillance-based evidence: Elimination of schistosomiasis as a public health problem in the Peoples’ Republic of China. Infect. Dis. Poverty.

[10] Wang W., Bergquist R., King C.H., Yang K. (2021). Elimination of schistosomiasis in China: Current status and future prospects. PLoS Negl. Trop. Dis..

[11] Utzinger J., Becker S.L., van Lieshout L., van Dam G.J., Knopp S. (2015). New diagnostic tools in schistosomiasis. Clin. Microbiol. Infect..

[12] Lindholz C.G., Favero V., Verissimo C.M., Candido R.R.F., de Souza R.P., Dos Santos R.R., Morassutti A.L., Bittencourt H.R., Jones M.K., St Pierre T.G. (2018). Study of diagnostic accuracy of Helmintex, Kato-Katz, and POC-CCA methods for diagnosing intestinal schistosomiasis in Candeal, a low intensity transmission area in northeastern Brazil. PLoS Negl. Trop. Dis..

[13] Gray D.J., Ross A.G., Li Y.S., McManus D.P. (2011). Diagnosis and management of schistosomiasis. BMJ.

[14] LoVerde P.T. (2019). Schistosomiasis. Adv. Exp. Med. Biol..

[15] Lv C., Deng W., Wang L., Qin Z., Zhou X., Xu J. (2022). Molecular techniques as alternatives of diagnostic tools in China as schistosomiasis moving towards elimination. Pathogens.

[16] Hinz R., Schwarz N.G., Hahn A., Frickmann H. (2017). Serological approaches for the diagnosis of schistosomiasis—A review. Mol. Cell Probes.

[17] Chen C., Guo Q., Fu Z., Liu J., Lin J., Xiao K., Sun P., Cong X., Liu R., Hong Y. (2021). Reviews and advances in diagnostic research on *Schistosoma japonicum*. Acta Trop..

[18] Zhang J.F., Xu J., Bergquist R., Yu L.L., Yan X.L., Zhu H.Q., Wen L.Y. (2016). Development and application of diagnostics in the national schistosomiasis control programme in the People’s Republic of China. Adv. Parasitol..

[19] Zhou Y., Chen Y., Jiang Q. (2021). History of human schistosomiasis (bilharziasis) in China: From discovery to elimination. Acta Parasitol..

[20] Wang X.Y., Yang K. (2016). Serological diagnosis methods of schistosomiasis japonica at different prevalence: A meta-analysis. Chin. J. Schisto. Control.

[21] Zhu H., Yu C., Xia X., Dong G., Tang J., Fang L., Du Y. (2010). Assessing the diagnostic accuracy of immunodiagnostic techniques in the diagnosis of schistosomiasis japonica: A meta-analysis. Parasitol. Res..

[22] Wang W., Li Y., Li H., Xing Y., Qu G., Dai J., Liang Y. (2012). Immunodiagnostic efficacy of detection of *Schistosoma japonicum* human infections in China: A meta analysis. Asian Pac. J. Trop. Med..

[23] Liu J. (2011). The role of the funnel plot in detecting publication and related biases in meta-analysis. Evid. Based Dent..

[24] Hartzes A.M., Morgan C.J. (2019). Meta-analysis for diagnostic tests. J. Nucl. Cardiol..

[25] Zhou X.N., Guo J.G., Wu X.H., Jiang Q.W., Zheng J., Dang H., Wang X.H., Xu J., Zhu H.Q., Wu G.L. (2007). Epidemiology of schistosomiasis in the People’s Republic of China, 2004. Emerg. Infect. Dis..

[26] Cao C.L., Zhang L.J., Deng W.P., Li Y.L., Lv C., Dai S.M., Feng T., Qin Z.Q., Duan L.P., Zhang H.B. (2020). Contributions and achievements on schistosomiasis control and elimination in China by NIPD-CTDR. Adv. Parasitol..

[27] Peng X.H., Hou C.S., Shi C.C. (1982). Evaluating the value of indirect hemagglutination assay for the field diagnosis of schistosomiasis japonica. Sichuan Med..

[28] Guan W.H., Yuan H.C., Zhao G.M., Yu J.M., Yang Q.J. (1999). Survey of schistosomiasis epidemic in dam-circle marsh region. Chin. J. Public Health.

[29] Wu Z.W., Liu Z.C., Yang G.F. (2000). Reliability of application of IHA method for determine chemotherapy targets of schistosomiasis in moderately endemic areas of lake region. Chin. J. Schist. Control.

[30] Li J.H., Wang T.P., Xiao X., Wu W.D., Lv D.B., Fang G.R., Cai W., Zheng J., Xu J., Wang R.R. (2002). Cost-effectiveness analysis on different schistosomiasis case screen methods in hypo-endemic area. J. Pract. Parasit. Dis..

[31] Li J.H., Wang T.P., Xiao X., Wu W.D., Lv D.B., Fang G.R., Cai W., Zheng J., Xu J., Wang R.R. (2002). Cost-effectiveness analysis on different schistosomiasis case screen methods in hypo-endemic area. Chin. J Schist. Control..

[32] Sun L.P., Hong Q.B., Zhou X.N., Huang Y.X., Wu F., Zhang Y.P., Yang G.J. (2002). Field evaluation of fraction antigen of SEA applied in screening of schistosomiasis. Chin. Parasit. Dis Control..

[33] Song H.T., Liang Y.S., Dai J.R., Li H.J., Ji C.S., Shen X.H., Li L.G., Yin F. (2003). Cost-effectiveness of three immunoassays for diagnosis of schistosomiasis in lower endemic area. Chin. J. Schist. Control.

[34] Zhang J.J., Xu L., Song H.T. (2004). Application of DDIA for screening schistosomiasis in high-risk populations. J. Trop. Dis. Parasitol..

[35] Fang W., Gan Z.M., Dong P.H., Yang T.L., Chen F., Luo B.R., Qiu Z.L. (2004). Field application of dipstick dye immunoassay in schistosomiasis-endemic areas in Yunnan Province. Chin. J. Schist. Control.

[36] Dai J.R., Zhu Y.C., Liang Y.S., Zhao S., Li H.J., Xu Y.L., Hua W.Q., Cao G.Q., Xu M. (2004). Study on scheme for screening schistosomiasis in low endemic areas. Chin. J. Schist. Control.

[37] Zhu Y.C., He W., Dai J.R., Xu M., Liang Y.S., Tang J.X., Hua W.Q., Cao G.Q., Chen H.G., Lou P.A. (2004). Application of dipstick dye immunoassay (DDIA) kit on detection of schistosomiasis japonica on large scale in endemic areas. Chin. J. Schist. Control.

[38] Liang Y.S., Zhu Y.C., Dai J.R., He W., Xu M., Li Y.L., Wang S.W., Tang J.X., Hua W.Q., Li H.J. (2005). Field application of dipstick dye immunoassay (DDIA) kit for detecting schistosomiasis in mountainous endemic regions in Yunnan Province. Chin. J. Schist. Control.

[39] Wang S.W., Yang Z., Yin G.L., Li Y.L., Yang J., Zhao J.B., Luo B.R., Zuo X.F., Zou H.M., Zhang J.P. (2005). Application of antibody-based ELISA for detection of schistosomiasis in highly endemic regions. Parasit. Infect. Dis..

[40] Xiao X., Wang T., Ye H., Qiang G., Wei H., Tian Z. (2005). Field evaluation of a rapid, visually-read colloidal dye immunofiltration assay for *Schistosoma japonicum* for screening in areas of low transmission. Bull. World Health Organ..

[41] Wang Q.Z., Wang F.F., Yin X.M., Zhu L., Zhang G.H., Fang G.R., Wang T.P., Xiao X., Jiang Q.W. (2006). Evaluation of screening effects of ELISA and IHA techniques in different epidemic areas of schistosomiasis. J. Trop. Dis. Parasitol..

[42] Wu Y.L., Cheng M., Meng W., Li H.X. (2006). Comparison between effect of two schistosomiassi diagnostic kit application to the multimountain area in Yunnan Province. BMC J..

[43] Bao J.G., Qiang G.X., Deng Y.J., Zhang R., Chen Y. (2006). Comparison of four diagnostic assays for the field detection of schistosomiasis. J. Trop. Dis. Parasitol..

[44] Sun L.P., Hong Q.B., Huang Y.X., Liang Y.S., Xu M., Zhang L.H., Gao Y., Zhou M., Yang K., Zhu Y.C. (2004). Comparison of two immunoassays for schistosomiaisis diagnosis in the field. Chin. J. Schist. Control.

[45] Chen S.R., Chen F., Zhou X.N., Li H.J., Stenmann P.J., Yang Z., Li Y.L. (2007). Comparison of aetiological and serological diagnosis methods in schistosomiasis mountainous endemic area. Parasitol. Infect. Dis..

[46] He W., Zhu Y.C., Liang Y.S., Dai J.R., Xu M., Tang J.X., Cao G.Q., Hua W.Q., Li Y.L., Yang Z. (2007). Comparison of stool examination and immunodiagnosis for schistosomiasis. Chin. J. Schist. Control.

[47] Xu J., Chen N.G., Feng T., Wang E.M., Wu X.H., Chen H.G., Wang T.P., Zhou X.N., Zheng J. (2007). Effectiveness of routinely used assays for the diagnosis of schistosomiasis japonica in the field. Chin. Parasitol. Parasit. Dis..

[48] Yang Z., Yin G.L., Fan C.Z., Luo B.R., Liu Y.H., Duan Y.C., Cui Y.H., Yang Y.N., Sun H.Y., Wang S.W. (2007). Effect of gold labeling immunoassay for diagnosis of schistosomiasis. Parasitol. Infect. Dis..

[49] Zhou Y.B., Yang M.X., Wang Q.Z., Zhao G.M., Wei J.G., Peng W.X., Jiang Q.W. (2007). Field comparison of immunodiagnostic and parasitological techniques for the detection of Schistosomiasis japonica in the People’s Republic of China. Am. J. Trop. Med. Hyg..

[50] Yu J.M., de Vlas S.J., Jiang Q.W., Gryseels B. (2007). Comparison of the Kato-Katz technique, hatching test and indirect hemagglutination assay (IHA) for the diagnosis of *Schistosoma japonicum* infection in China. Parasitol. Int..

[51] Wang X.H., Zhou X.N., Li Y.L., Lv S., Li L.H., Jia T.W., Chen S.R., Yang Z., Fang W., Chen F. (2007). Evaluation of two tests for detecting *Schistosoma japonicum* infection using a Bayesian approach. Chin. J. Health Stat..

[52] Lin D.D., Liu Y.M., Hu F., Tao B., Wang X.M., Zuo X.X., Li J.Y., Wu G.L. (2008). Evaluation on application of common diagnosis methods for schistosomiasis japonica in endemic areas of China I Evaluation on estimation of prevalence of Schistosoma japonicum infection by IHA screening method. Chin. J. Schist. Control.

[53] Zhou Y.B., Yang M.X., Tao P., Jiang Q.L., Zhao G.M., Wei J.G., Jiang Q.W. (2008). A longitudinal study of comparison of the Kato-Katz technique and indirect hemagglutination assay (IHA) for the detection of schistosomiasis japonica in China, 2001–2006. Acta Trop..

[54] Lin D.D. (2008). Evaluation of the Performance of Commonly Used Diagnostic Assays for the Field Detection of Schistosomiasis Japonica in China.

[55] Lin D.D., Xu J.M., Zhang Y.Y., Liu Y.M., Hu F., Xu X.L., Li J.Y., Gao Z.L., Wu H.W., Kurtis J. (2008). Evaluation of IgG-ELISA for the diagnosis of *Schistosoma japonicum* in a high prevalence, low intensity endemic area of China. Acta Trop..

[56] Jiang B., Zhou Y.D., Meng Q.Y., Luo Q.L., Shen J.L. (2009). Study on IgY immunoglobulin-based double antibody sandwich ELISA for schistosomiaisis diagnosis in the field. J. Trop. Med. Parasitol..

[57] Zhang S.W., Cheng B., Qu H.J., Chen Z.M., Zou Q., Chu L.P., Zhang L., He H.R., Tang S.H., Huang X.P. (2010). Validity evaluation of dipstick dye immuno-assay (DDIA) for screening in low endemic areas of schistosomiasis. Chin. J. Schist. Control.

[58] Wang P., Ren C.P., Wang T.P., Shen J.J. (2010). Evaluation of recombinant 29 000 extra membranous protein for the ummunodiagnosis of schistosomiasis japonica. Chin. J. Parasitol. Parasit. Dis..

[59] Xu J., Feng T., Lin D.D., Wang Q.Z., Tang L., Wu X.H., Guo J.G., Peeling R.W., Zhou X.N. (2011). Performance of a dipstick dye immunoassay for rapid screening of *Schistosoma japonicum* infection in areas of low endemicity. Parasit. Vectors.

[60] Zhou X.N., Xu J., Chen H.G., Wang T.P., Huang X.B., Lin D.D., Wang Q.Z., Tang L., Guo J.G., Wu X.H. (2011). Tools to support policy decisions related to treatment strategies and surveillance of Schistosomiasis japonica towards elimination. PLoS Negl. Trop. Dis..

[61] Sun C.S., Wang F.F., Wang Y., Zhou L., Yin X.M., Wang Q.Z., Zhang L.S., Wang E.M., Zhang S.Q. (2013). Effectiveness of an indirect hemagglutination assay kit at diagnosing schistosomiasis in the field. J. Parasit. Biol..

[62] Hu F., Li Z.J., Li Y.F., Yuan M., Xie S.Y., Liu Y.M., Li J.Y., Gao Z.L., Pu Y., Wang J.M. (2016). Study on cut-off value of IHA method for schistosomiasis diagnosis in different endemic areas. Chin. J. Schist. Control.

[63] Xing W., Yu X., Feng J., Sun K., Fu W., Wang Y., Zou M., Xia W., Luo Z., He H. (2017). Field evaluation of a recombinase polymerase amplification assay for the diagnosis of *Schistosoma japonicum* infection in Hunan province of China. BMC Infect. Dis..

[64] Weerakoon K.G., Gobert G.N., Cai P., McManus D.P. (2015). Advances in the diagnosis of human schistosomiasis. Clin. Microbiol. Rev..

[65] Zhang S.Q., Sun C.S., Wang M., Lin D.D., Zhou X.N., Wang T.P. (2016). Epidemiological features and effectiveness of schistosomiasis control programme in lake and marshland region in the People’s Republic of China. Adv. Parasitol..

[66] Shi L., Li W., Wu F., Zhang J.F., Yang K., Zhou X.N. (2016). Epidemiological features and control progress of schistosomiasis in waterway-network region in the People’s Republic of China. Adv. Parasitol..

[67] Liu Y., Zhou Y.B., Li R.Z., Wan J.J., Yang Y., Qiu D.C., Zhong B. (2016). Epidemiological features and effectiveness of schistosomiasis control programme in mountainous and hilly region of the People’s Republic of China. Adv. Parasitol..

[68] He P., Gordon C.A., Williams G.M., Li Y., Wang Y., Hu J., Gray D.J., Ross A.G., Harn D., McManus D.P. (2018). Real-time PCR diagnosis of *Schistosoma japonicum* in low transmission areas of China. Infect. Dis. Poverty.

[69] Fung M.S., Xiao N., Wang S., Carlton E.J. (2012). Field evaluation of a PCR test for *Schistosoma japonicum* egg detection in low-prevalence regions of China. Am. J. Trop. Med. Hyg..

[70] Lier T., Johansen M.V., Hjelmevoll S.O., Vennervald B.J., Simonsen G.S. (2008). Real-time PCR for detection of low intensity *Schistosoma japonicum* infections in a pig model. Acta Trop..

[71] Xu J., Guan Z.X., Zhao B., Wang Y.Y., Cao Y., Zhang H.Q., Zhu X.Q., He Y.K., Xia C.M. (2015). DNA detection of *Schistosoma japonicum*: Diagnostic validity of a LAMP assay for low-intensity infection and effects of chemotherapy in humans. PLoS Negl. Trop. Dis..

[72] Xu J., Rong R., Zhang H.Q., Shi C.J., Zhu X.Q., Xia C.M. (2010). Sensitive and rapid detection of *Schistosoma japonicum* DNA by loop-mediated isothermal amplification (LAMP). Int. J. Parasitol..

[73] Wang C., Chen L., Yin X., Hua W., Hou M., Ji M., Yu C., Wu G. (2011). Application of DNA-based diagnostics in detection of schistosomal DNA in early infection and after drug treatment. Parasit. Vectors.

[74] Song Z., Ting L., Kun Y., Wei L., Jian-Feng Z., Li-Chuan G., Yan-Hong L., Yang D., Qing-Jie Y., Hai-Tao Y. (2018). Establishment of a recombinase-aided isothermal amplification technique to detect *Schistosoma japonicum* specific gene fragments. Chin. J. Schisto. Control.

[75] Ye Y.Y., Zhao S., Liu Y.H., Zhang J.F., Xiong C.R., Ying Q.J., Yang K. (2021). Establishment of a nucleic acid dipstick test for detection of *Schistosoma japonicum* specific gene fragments based on the recombinase-aided isothermal amplification assay. Chin. J. Schist. Control.

[76] Zhao S., Liu Y.H., Li T., Li W., Zhang J.F., Guo L.C., Ying Q.J., Yang H.T., Yang K. (2019). Rapid detection of *Schistosoma japonicum* specific gene fragment by recombinase aided isothermal amplification combined with fluorescent probe. Chin. J. Parasitol. Parasit. Dis.

[77] Sun K., Xing W., Yu X., Fu W., Wang Y., Zou M., Luo Z., Xu D. (2016). Recombinase polymerase amplification combined with a lateral flow dipstick for rapid and visual detection of *Schistosoma japonicum*. Parasit. Vectors.

[78] Deng W., Wang S., Wang L., Lv C., Li Y., Feng T., Qin Z., Xu J. (2022). Laboratory evaluation of a basic recombinase polymerase amplification (RPA) assay for early detection of *Schistosoma japonicum*. Pathogens.

[79] WHO Ending the Neglect to Attain the Sustainable Development Goals: A Road Map for Neglected Tropical Diseases 2021–2030. https://www.who.int/publications/i/item/9789240010352.

[80] WHO WHO Guideline on Control and Elimination of Human Schistosomiasis. https://www.who.int/publications/i/item/9789240041608.

[81] Chen J., Bergquist R., Zhou X.N., Xue J.B., Qian M.B. (2019). Combating infectious disease epidemics through China’s Belt and Road Initiative. PLoS Negl. Trop. Dis..

[82] Wang X.Y., He J., Juma S., Kabole F., Guo J.G., Dai J.R., Li W., Yang K. (2019). Efficacy of China-made praziquantel for treatment of schistosomiasis haematobium in Africa: A randomized controlled trial. PLoS Negl. Trop. Dis..

[83] Xing Y.T., Dai J.R., Yang K., Jiang T., Jiang C.G., Mohammed S.J., Kabole F., Yang K., Mehlhorn H. (2021). *Bulinus* snails control by China-made niclosamide in Zanzibar: Experiences and lessons. Sino-African Cooperation for Schistosomiasis Control in Zanzibar.

[84] Zhang L.J., Mwanakasale V., Xu J., Sun L.P., Yin X.M., Zhang J.F., Hu M.C., Si W.M., Zhou X.N. (2020). Diagnostic performance of two specific *Schistosoma japonicum* immunological tests for screening *Schistosoma haematobium* in school children in Zambia. Acta Trop..

[85] Zhu Y.C., Socheat D., Bounlu K., Liang Y.S., Sinuon M., Insisiengmay S., He W., Xu M., Shi W.Z., Bergquist R. (2005). Application of dipstick dye immunoassay (DDIA) kit for the diagnosis of schistosomiasis mekongi. Acta Trop..

[86] Hua H.Y., Wang W., Cao G.Q., Tang F., Liang Y.S. (2013). Improving the management of imported schistosomiasis haematobia in China: Lessons from a case with multiple misdiagnoses. Parasit. Vectors.

[87] Zhu Y.C., Hassen S., He W., Cao G.Q. (2006). Preliminary study on detection of schistosomiasis mansoni with dipstick dye immunoassay (DDIA) kit. Chin. J. Schist. Control.

